# Review of "Glossary of Biotechnology and Nanobiotechnology Terms" by Kimball Nill

**DOI:** 10.1186/1475-925X-6-5

**Published:** 2007-01-31

**Authors:** Ming Jiang

**Affiliations:** 1 LMAM, School of Mathematical Sciences, Peking University, Beijing 100871, China

Biotechnology is now not only the field of professionals but also frequently shows up in newspapers and popular magazines for the general public with its advanced and diverse applications in fields such as food industry and healthcare that affect the daily life and future of human being. With the rapid expanding and evolving of biotechnology, new terms are entering the nomenclature at a rapid pace (from the preface), though they are not easily understandable except for a small group of experts. It is important for scientists of other branches to keep abreast of the latest terminology and necessary for non-professionals to understand buzzwords such as "Apo A-1 Milano", "CD95 Protein", "FRET", "hedgehog signaling pathway", "lysophosphatidylethanolamine". "Glossary of Biotechnology and Nanobiotechnology Terms, Fourth Edition", is "*a handy reference designed for people with little or no training in the biological and chemical sciences*" (from the back cover) written for the aforementioned purposes.

The book provides the definitions of biotechnolgy and nanobiotechnology terminology which includes terms from the fields of biology, biochemistry, chemistry, and nanotechnology. The current book is a result of the author's effort of continuing improvement over a decade since the first edition published in 1993. The current edition contains 402 text pages, more than two and half times of the first edition. In this edition, the author has added "nanotech" terms relevant to biotechnology to the glossary (from the preface).

The book is intended for "*anyone working directly or indirectly with those pioneering the frontiers of modern biology*" (from the back cover). The author has written the book for readers without necessarily holding advanced degrees in biochemistry or molecular biology and made certain compromise between absolute scientific rigor and definitions based on analogy, with the inherent possibility of oversimplification (from the preface). The cross reference after each item is extensive and provides the logical connections with other items for readers to explore relevant topics.

Some comments about the book are in the following. The cross-references would be ideal in a hyperlink form such as an online format or in a companying CDROM. I found by Google that an online format is available for its earlier 2^nd ^edition [1]. However, the content there is not as updated as the current 4^th ^edition. Definitions can be more illustrative with color figures. especially for those items about cell, protein or DNA structures. Moreover, I do not agree with the statement on the back cover that the book "*allows one to follow a reference chain that enhances clarity right down to a high school level*." Extra efforts and references are required to fully understand the analogies and examples. Finally, let me quote one interesting comment by Eleanor Randall in the review of the same book: "*As I began this review, I needed a clearer definition of nanobiotechnology and its relationship with bionanotechnology. Interestingly, the title term nanobiotechnology was not included in The Glossary and, in the preface, one finds the term bionanotechnology used*." [2]

The book will help one keep current with the biotechnology and nanotechnology terminology, communicate successfully with those working on the cutting-edge of modern science and enter interdisciplinary collaborations. This book is recommended to scientists, engineers, attorneys, government workers, lobbyists, venture capitalists, and university tech transfer staff, especially for personnel with no advanced training in biological and chemical sciences to understand concepts and buzzwords that are indispensable to their work. Nevertheless, biotechnologist, who is probably only an expert in one of the diverse areas of biotechnology, and advanced students in one of the many biotechnology fields, may find it useful.
